# Genome-Wide Identification of GH17s Family Genes and Biological Function Analysis of SlA6 in Tomato

**DOI:** 10.3390/plants13172443

**Published:** 2024-09-01

**Authors:** Da Chen, Zaohai Zeng, Canye Yu, Huimin Hu, Yuxiang Lin, Caiyu Wu, Yinghua Yang, Qiuxiang Zhong, Xinyue Zhang, Caihong Huang, Yiwen Yao, Zhengkun Qiu, Xiaomin Wang, Rui Xia, Chongjian Ma, Riyuan Chen, Yanwei Hao, Hongling Guan

**Affiliations:** 1 Key Laboratory of Horticultural Crop Biology and Germplasm Innovation in South China, Ministry of Agriculture, College of Horticulture, South China Agricultural University, Guangzhou 510642, China; d.chen@stu.scau.edu.cn (D.C.); zengzh@scau.edu.cn (Z.Z.); cyyu@stu.scau.edu.cn (C.Y.); huhuimin1013@163.com (H.H.); lyxtulip@163.com (Y.L.); wucaiyu1995@163.com (C.W.); qqs2961180779@163.com (Y.Y.); zqx15990173181@163.com (Q.Z.); xinyue_z2021@163.com (X.Z.); 18312541354@163.com (C.H.); prometheus03@163.com (Y.Y.); qiuzhengkun@scau.edu.cn (Z.Q.); rxia@scau.edu.cn (R.X.); rychen@scau.edu.cn (R.C.); 2 Guangdong Provincial Key Laboratory of Utilization and Conservation of Food and Medicinal Resources in Northern Region, School of Biology and Agriculture, Shaoguan University, Shaoguan 512005, China; chjma@sgu.edu.cn; 3 Key Laboratory of Plant Hormones and Development Regulation of Chongqing, School of Life Sciences, Chongqing University, Chongqing 400044, China; 4 Key Laboratory of Mountain Biodiversity Conservation in Guangxi Universities, College of Biological and Pharmaceutical Sciences, Yulin Normal University, Yulin 537000, China; wxmin99@163.com

**Keywords:** glycoside hydrolases, GH17, SlA6, pollen development, tomato

## Abstract

Glycoside hydrolases (GHs), enzymes that break down glycosidic bonds in carbohydrates and between carbohydrates and non-carbohydrates, are prevalent in plants, animals, microorganisms, and other organisms. The tomato is a significant crop that contains the GH17 gene family. However, its role in tomatoes has yet to be fully investigated. In this study, we identified 43 GH17 genes from the tomato genome, distributed unevenly across 12 chromosomes. We further analyzed their gene structure, phylogenetic relationships, promoter elements, and expression patterns. The promoter element analysis indicated their potential roles in response to biotic and abiotic stresses as well as phytohormone effects on growth and development. The expression studies across different tomato tissues revealed that 10 genes were specifically expressed in floral organs, with SlA6 prominently expressed early during bud formation. By using CRISPR/Cas9 gene-editing technology, *SlA6* knockout plants were generated. Phenotypic characterization showed that pollen viability, pollen tube germination, fruit weight, and seed number were significantly reduced in the *Sla6* mutant, but the soluble solids content (TSS) was significantly higher in the *Sla6* mutant, suggesting that SlA6 affects pollen development and fruit quality.

## 1. Introduction

Glycoside hydrolases (GHs) are enzymes that hydrolyze glycosidic bonds, playing crucial roles in the degradation and synthesis of sugars and glycoconjugates in organisms. Classified by the Carbohydrate-Active enZymes database (CAZy), GHs are categorized into 189 families (Glycoside Hydrolase family), numbered from GH1 to GH189, with members within each family sharing high structural similarities. The GH17 family includes various enzymes, such as endo-β-1,3-glucanases, exo-β-1,3-glucanases, β-1,3-1,4-glucanases, and β-1,3-glucosyltransferases, with endo-β-1,3-glucanases being the predominant members [1,2].

GH17 family enzymes have diverse functions in plants. In barley and oats seeds, β-1,3-1,4-glucanases hydrolyzed β-glucans in the endosperm cell walls, which are crucial for seed germination, highlighting the pivotal roles of GH17 genes in seedling germination [3,4]. In Arabidopsis, GH17 genes have been linked to the formation of intercellular bridges, indicating their potential role in cell-to-cell communication [5,6]. The overexpression of GH17 genes in poplar promotes axillary bud differentiation, which is regulated by gibberellins, indicating their involvement in hormone-mediated plant nutritional growth [7]. In sorghum, GH17 genes are involved in polysaccharide degradation. In grapes, their expression varies significantly during fruit and dormant bud development, highlighting their importance in fruit maturation [8,9]. Additionally, GH17 family enzymes play crucial roles in the defense against biotic and abiotic stresses in plants. In Panax notoginseng, a *GH17* homolog known as *PnGlu1* enhanced resistance to Fusarium in tobacco, underscoring its vital role in plant defense mechanisms [10]. Proteomic analyses indicated that GH17s responded to the salt stress in ginseng leaves. The overexpression of GH17 in Arabidopsis increased the salt tolerance of transgenic plants. In rice, the endo-β-1,3-glucanase levels increased under ABA and drought conditions, indicating the role of GH17 in abiotic stress responses [11,12].

Moreover, GH17 family enzymes play a crucial role in plant reproductive development. The GH17 genes in *Brassica oleracea* exhibit variable expression levels and are notably downregulated in pollen tissues (BraA04g008040, BraA07g009320, BraA01g030220, BraA03g040850, BraA10g020080, and BraA05g038340), indicating their importance in pollen development [13]. The overexpression of endo-β-1,3-glucanase genes in tobacco leads to pollen-coat defects and male sterility [14]. β-1,3-glucanase’s activity is also correlated with the absence of sporopollenin in lilies [15]. β-1,3-glucanases are also reported to participate in the reproductive development of both monocots and dicots [16]. The differential β-1,3-glucanase activity seen in fertile and sterile wheat lines suggests their crucial role in male sterility [17]. In Arabidopsis, β-1,3-glucan is essential for pollen wall growth [18]. *AtA6*, a gene specifically expressed in tapetal cells, encodes an endo-β-1,3-glucanase belonging to the GH17 family [19]. In *Arabidopsis*, *A6* acts as a crucial downstream target gene of MYB80 and significantly contributes to the breakdown of tapetal cells during anther development [20].

Our previous research showed that *SlA6*, the tomato homolog of *AtA6*, was regulated by SlHB8, whose overexpression led to abnormal pollen formation in the tomato [21]. In this study, we identified 43 GH17 genes from the tomato genome. Expression studies across different tomato tissues revealed that *SlA6* was prominently expressed early during bud formation. The loss of the function mutant of SlA6 by using CRISPR/Cas9 gene-editing technology showed reduced pollen activity and pollen tuber germination rates, as well as fewer seeds in the fruit, indicating its important role in pollen development. These findings suggest a potential role of *SlA6* in tomato pollen development and underscore the importance of further studies on SlA6 and the GH17 family.

## 2. Results

### 2.1. Identification of GH17 Family Members and Analysis of Their Physicochemical Properties

In *Arabidopsis*, the GH17 subfamily has 51 members. In the tomato genome, 43 *SlGH17* genes were identified by comparing and validating the GH17 structural domain (PF00332). Based on their positions in the genome, they were named *SlGH17-1* to *SlGH17-43*, with *SlGH17-43* being SlA6. The physicochemical analyses showed that the lengths of the tomato GH17 genes varied slightly, with the amino acids ranging from 336 to 526, molecular weights from 37.45 to 57.62, and theoretical pI values from 4.84 to 9.42. The stability coefficients of the *SlGH17s* ranged from 26 to 46, with 82% having coefficients below 40, indicating that most SlGH17s were stable proteins. The aliphatic amino acid index ranged from 76 to 107.3, indicating their high thermal stability. Additionally, 82% of the members had negative hydrophilicity coefficients, while the rest had positive values, indicating that most SlGH17 proteins were hydrophilic, and a few were hydrophobic (Table 1).

**Figure 1:**
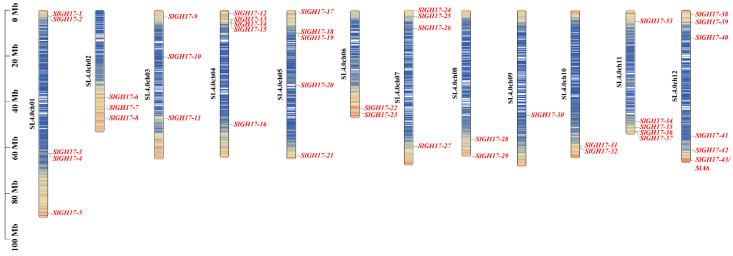
Chromosome localization of SlGH17 genes. Note: SlA6 is SlGH17-43. Note: Blue-white-red represents an increasing density of genes. Red represents gene names and black represents chromosome names.

The GH17 genes in tomatoes are distributed across 12 chromosomes, as shown in Figure 1. Only one gene appears on chromosome 9, whereas six genes were found on chromosome 12. Several GH17 genes tend to cluster together, indicating that these groups may have evolved new family members through tandem replication events. Such a clustering suggests that these genes could share similar functions, offering valuable insights for future research into their roles.

### 2.2. Protein Motifs, Conserved Domains, and Gene Structure Analysis of GH17 Family Members

**Figure 2:**
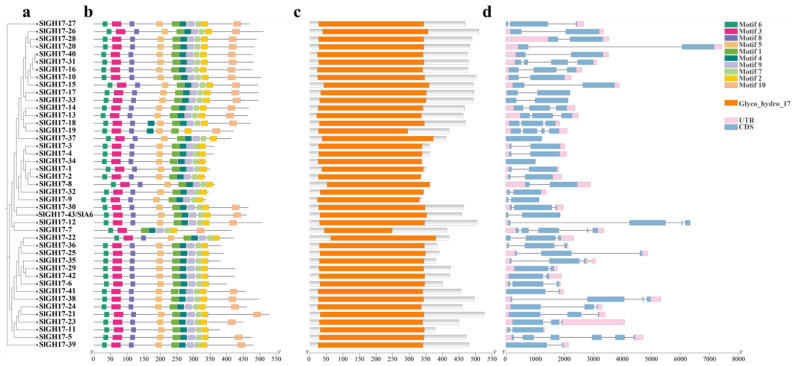
The gene structure, protein motif, and conserved protein domains of SlGH17s based on their evolutionary relationship. Note: (**a**) Evolutionary analysis of SlGH17s; (**b**) conserved motif visualization of SlGH17s; (**c**) conserved domain visualization; (**d**) gene structure visualization. CDS: coding sequences; UTR: untranslated regions.

The conserved motifs detection, using the MEME online tool, revealed that all SlGH17 proteins contain motifs 6, 3, 8, 5, 1, 4, 9, and 2, except SlGH17-7. Some SlGH17s also include motif 10, and most have motif 7 (Figure 2b). Batch CD-search and SMART domain analysis confirmed that all the SlGH17 proteins possess the conserved GH17 structural domain (PF00332), consistent with the GH17 family domains in *Arabidopsis* (Figure 2c). Gene structural analysis showed minimal variations in the number of exons (1–6) and introns (0–5) in the *SlGH17s’* genomic sequences (Figure 2d, Table S1).

### 2.3. Evolutionary and Covariance Analyses of GH17 Family Members

**Figure 3:**
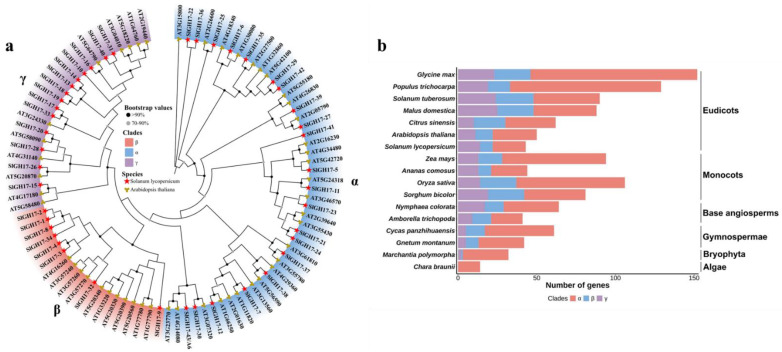
Phylogenetic and family numbers analysis of GH17 genes in different plant species. Note: (**a**) ML phylogenetic evolutionary trees of SlGH17s and AtGH17s, generated by using IQ-tree software (version: 2.3.6). All GH17 family genes were grouped into three subfamilies, named α, β, and γ. The α subfamilies are colorized by the blue zone; β subfamilies are colorized by the red zone. The purple zone is the γ subfamilies. The red five-pointed star represents tomato GH17s, and the yellow five-pointed star represents Arabidopsis GH17s. (**b**) Analysis of the GH17 family members in different plant species.

The phylogenetic tree analysis revealed that GH17s in tomatoes are categorized into three subfamilies, similar to *Arabidopsis*: the α subfamily with 21 members, the β subfamily with 8, and the γ subfamily with 14, totaling 43 members. The *SlA6* gene belongs to the α subfamily (Figure 3a). We analyzed the number of GH17 members in different species and found that algae possess fewer GH17 family members exclusively within the α subfamily (Figure 3b). As the species evolved, an increase in both amplification and branching among the GH17 family members occurred; dicotyledonous plants possess the highest count of these family members, covering the α, β, and γ subfamilies and, in particular, have a predominant number in the α group (Figure 3b, Table S2). The covariance analysis revealed gene duplication events within the tomato species as well as inter-species duplicate occurrences for the GH17 genes (Figure S2).

### 2.4. Cis-Acting Elements Analysis of the Promoter Region of GH17 Family Members

**Figure 4:**
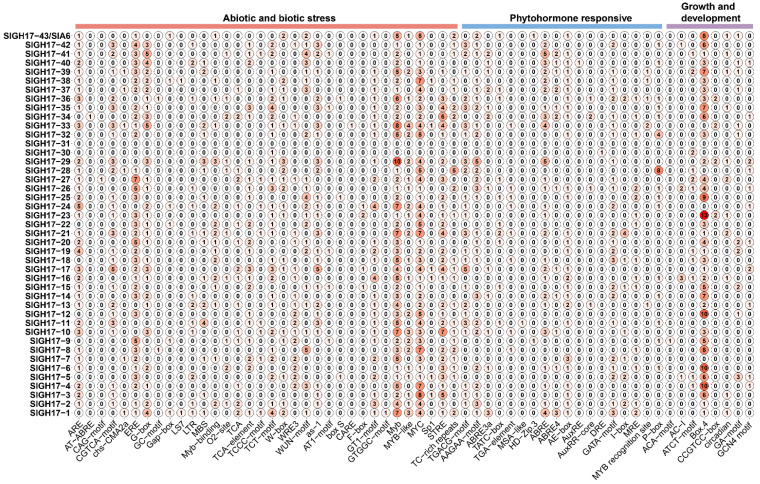
Cis-acting elements Analysis of the promoter region of SlGH17s. Note: The value in the circle represents the number of the cis-acting elements. The redder the color, the more cis-acting elements it contains.

To identify the promoter sequence of *SlGH17s*, we extracted a 2000 bp sequence upstream of the 5′ UTR from the tomato genomic data. We used the PlantCare website to identify cis-acting elements in the promoter. The analysis (Figure 4) revealed that this region contains numerous biotic and abiotic stress elements, followed by hormone-responsive elements. This suggests that GH17s were involved in responses to both biotic and abiotic stresses and also respond to hormones.

There are a large number of light-responsive elements such as ARE, AT~ABRE, CAG-motif, CGTCA-motif, chs-CMA2a, ERE, G-box, GC-motif, Gap-box, LS7, LTR, MBS, Myb-binding site, O2-site, TCA, TCA-element, TCCC-motif; anaerobic-inducing elements F-box; defense and stress response elements like TCT-motif, W box, WRE3, WUN-motif, and so on. The phytohormone response elements contained more abscisic acid response elements TGA-element, MSA-like, HD-Zip 3, ABRE, ABRE4, AE-box, followed by jasmonic acid response elements DRE, GATA-motif, I-box, MRE, MYB recognition site. In addition, there are also auxins and gibberellin response elements AuxRE, AuxRR-core, and P-box. Elements associated with growth and development are ACA-motif, AC-I, ATCT-motif, Box 4, CCGTCC-box, circadian, GA-motif, and GCN4_motif.

### 2.5. Expression Pattern Analysis of the SlGH17s Family Members in Tomato Tissues

**Figure 5:**
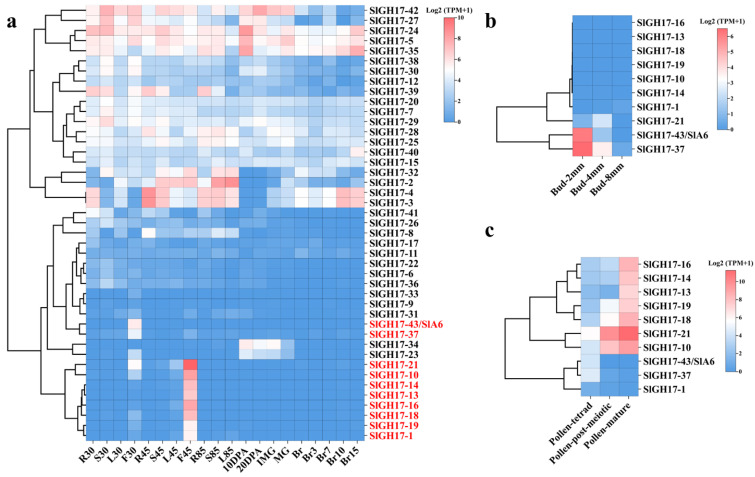
Expression analyses of SlGH17s in various tomato tissues. Note: (**a**) Expression analysis of SlGH17s in various Micro-Tom tomato tissues, 10 genes (in red color) showed the specifically highest expression in the flower bud at stage F30 and flower at stage F45, (**b**) expression analysis of 10 specifically expressed genes in anthers at different development stages of Micro-Tom tomato, (**c**) expression analysis of 10 specifically expressed genes in pollen at different development stages of Ailsa Craig tomato; samples F30, L30, R30, and S30 are flowers, leaves, roots, and stems collected 30 days after germination; samples F45, L45, R45, and S45 are flowers, leaves, roots, and stems collected 45 days after germination; samples L85, F85, and S85 are leaves, roots, and stems collected 85 days after germination; and samples 10DPA, 20DPA, IMG, MG, Br, Br3, Br7, Br10, and Br15 samples are fruit samples collected at 55, 65, 75, 80, 85, 92, 95, and 100 days after germination, respectively. The bud-2mm, bud-4mm, and bud-8mm samples are the tomato anthers collected when the length of the bud was 2 mm (microspore mother cell stage), 4 mm (tetrad stage), and 8 mm (mature pollen stage). Pollen-tetrad, pollen-meiotic, and pollen-mature are the pollen samples at different periods of pollen development.

The expression pattern of the *SlGH17* genes was analyzed using transcriptome data (BioProject: PRJNA559888) from various developmental stages of the Micro-Tom tomato. The data were normalized using TPM and further scaled from 0 to 1. The analysis revealed that *SlGH17* genes were expressed in all tomato tissues. Notably, 10 genes (in red color) showed the specifically highest expression in the flower bud at stage F30 and flower at stage F45, indicating their potential role in the development of floral organ tissues (Figure 5a).

To better understand the expression pattern of these 10 genes during flower development, we analyzed the transcriptomic data from different flower organs at various development stages. We found that SlGH17-43/A6 and SlGH17-37 were highly expressed in 2 mm buds and pollen-tetrad (Figure 5b,c), while SlGH17-10, SlGH17-13, SlGH17-14, SlGH17-16, SlGH17-19, SlGH17-18, and SlGH17-21 showed high expression levels in mature pollen (Figure 5c). This suggests that SlA6 may play a crucial role in the early stages of anther development (Figure 5b).

### 2.6. Expression Pattern Analysis of SlA6 during Tomato Flower Development and Response to Phytohormones

**Figure 6:**
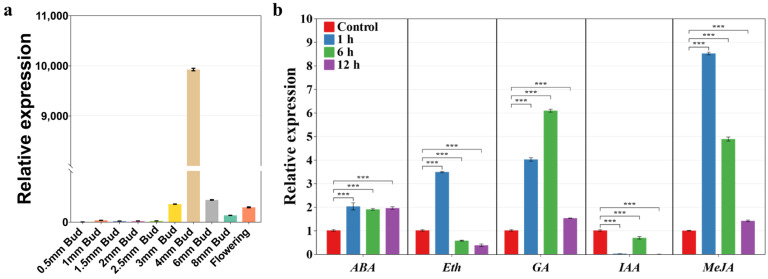
Expression pattern analyses of *SlA6* during tomato flower development and in response to phytohormones. Note: (**a**) Analysis of the expression pattern of SlA6 in tomato bud. (**b**) Analysis of the expression patterns of SlA6 in response to plant hormones in tomato seedlings. Sample names 0.5 mm, 1 mm, 1.5 mm, 2 mm, 2.5 mm, 3 mm, 4 mm, 6 mm, 8 mm, and on the day of flowering, are samples of the corresponding lengths of flower buds. ABA is abscisic acid, Eth is ethylene, GA is gibberellin, IAA stands for auxin, and MeJA is Methyl jasmonate. “***”, *p* < 0.001.

To understand the expression pattern of *SlA6* in tomato anthers at various developmental stages, the flower buds were categorized by length into the following groups: 0.5 mm, 1 mm, 1.5 mm, 2 mm, 2.5 mm, 3 mm, 4 mm, 6 mm, 8 mm, and on the day of flowering. We defined the anther development stages by combining the flower bud size with cell biological observations. Pollen was in the microspore mother cell stage for buds at ≤2 mm, tetrad stage for 3–4 mm buds, binuclear side stage for 5–6 mm buds, binuclear stage for 7–8 mm buds, with pollen maturation and anther cracking on the flowering day [21]. *SlA6* was expressed at all stages, with the highest expression occurring at the 4 mm bud stage and the lowest at the 0.5 mm bud stage (Figure 6). During flower development, *SlA6* expression initially increased and then decreased, suggesting its potential role in anther development, as supported by the transcriptome sequencing data (Figure 5).

To assess the response of *SlA6* to various phytohormones, seedlings of one month old were treated with MeJA, ABA, GA, Eth, and IAA for 1 h (h), 6 h, and 12 h. The results showed that after the ABA treatment, there was a significant upregulation of *SlA6* at all three time points. In contrast, the ethylene treatment caused a rapid increase in *SlA6* expression at 1 h followed by significant decreases at both 6 h and 12 h. After the GA treatment, *SlA6* expression was significantly upregulated at 1, 6, and 12 h. After the IAA treatment, *SlA6* expression was significantly downregulated at 1, 6, and 12 h. After the MeJA treatment, *SlA6* expression was significantly upregulated at 1, 6, and 12 h. This result indicated that *SlA6* responded strongly to phytohormones but in a different manner (Figure 6b).

### 2.7. Knocking Out of SlA6 Affects Pollen and Fruit Development in Tomato

**Figure 7:**
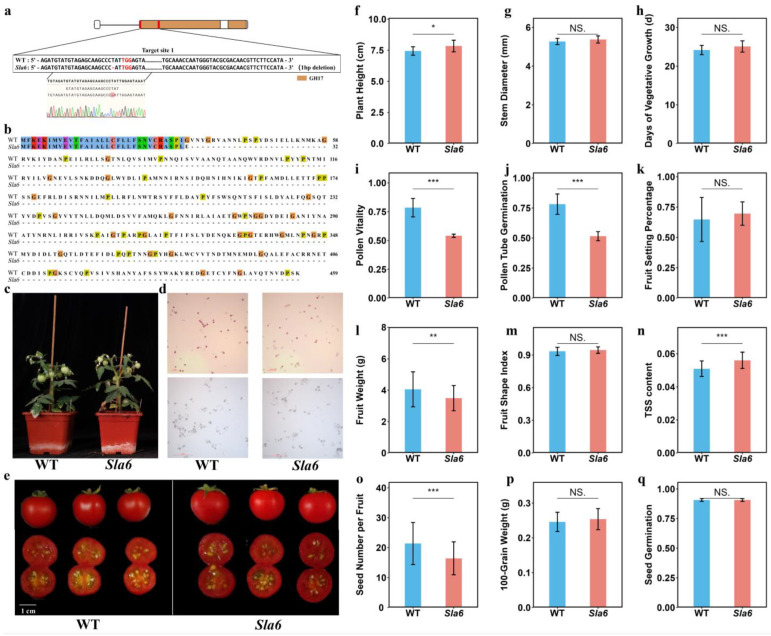
Gene editing types and phenotypic analyses of *SlA6* knockout plants. (**a**) Gene editing types of *SlA6* knockout plants. The white box represents the intron and the red part represents the target location. Red - is the deletion and red font is the CRISPR/Cas9 NGG recognition site. Different colored lines represent different bases. (**b**) amino acid sequence comparison between wild-type and *Sla6* mutant. The different colors represent jalview’s clustalx (version 2.16.0) color scheme. (**c**) Photo of wild-type and *Sla6* plants; (**d**) pollen staining of wild-type and *Sla6* plants; (**e**) fruit appearance of wild-type and *Sla6* plants; (**f**) height of wild-type and *Sla6* plants; (**g**) stem thickness of wild-type and *Sla6* plants; (**h**) days from germination to anthesis of wild-type and *Sla6* plants; (**i**) pollen vitality of wild-type and *Sla6* plants; (**j**) pollen tube gemination rate of wild-type and *Sla6* plants; (**k**) fruit set rate of wild-type and *Sla6* plants; (**l**) fruit weight of wild-type and *Sla6* plants; (**m**) fruit shape index of wild-type and *Sla6* plants; (**n**) TSS content of wild-type and *Sla6* plants; (**o**) seed number per fruit of wild-type and *Sla6* plants; (**p**) 100-grain weight of wild-type and *Sla6* plants; (**q**) seed germination rate of wild-type and *Sla6* plants. “*”, 0.01 ≤ *p* < 0.05; “**”, 0.001 ≤ *p* < 0.01; “***”, *p* < 0.001; “NS”, *p* ≥ 0.05.

Since *AtA6* affects the pollen development in *Arabidopsis*, we created a *SlA6* knockout mutant using CRISPR/Cas9 technology to determine whether *SlA6* regulates pollen development in tomatoes. Positive plants of the *SlA6* knockout mutant were identified by using PCR on DNA from both transgenic and wild-type plants (Figure S1). T0-transformed plants exhibiting the Cas9 PCR band were regarded as positive. We examined the mutation types at the target site in these T0 mutants. Homologous lines of these mutants of the T2 generation exhibited a single-base deletion of ‘T’ at target site 1. This deletion introduced a premature stop codon within the coding region (Figure 7a,b), leading to truncated translation and resulting in a loss of 427 amino acids from the protein sequence; this mutant was named *Sla6*.

The pollen viability and pollen tube germination rate of wild-type and *Sla6* mutant were examined. The results showed that the *Sla6* mutants had significantly lower pollen viability and pollen tube germination rates compared to the WT (Figure 7d,i,j). However, the fruit set rate of *Sla6* was not significantly different from the WT (Figure 7k).

Fruits of the wild-type and *Sla6* mutant at the Br+7 stage were collected for the fruit quality examination. The results revealed that the *Sla6* fruits had a significantly lower weight compared to the wild-type (WT) (Figure 7c,l). The fruit shape index of *Sla6* was not significantly different from the WT (Figure 7m). However, the total soluble solids (TSSs) content in *Sla6* was significantly higher than in the WT (Figure 7n). Additionally, the number of seeds per *Sla6* fruit was significantly reduced compared to the WT (Figure 7o). The 100-seed weight of *Sla6* seeds showed no significant difference from the WT (Figure 7p), and there was no difference in seed germination or the germination index between *Sla6* and the WT (Figure 7q). 

Additionally, the stem thickness of *Sla6* was not significantly different from that of the WT (Figure 7c,g). However, the plant height of *Sla6* was significantly higher compared to the WT (Figure 7f). The number of days from germination to anthesis for *Sla6* was also not significantly different from the WT (Figure 7h).

## 3. Discussion

This study analyzed the entire genome of the GH17 glycoside hydrolase superfamily in the tomato using bioinformatics methods. The GH17 subfamily comprises 43 members spread across 12 chromosomes, all containing conserved GH17 domains and several conserved motifs, though some gene motifs showed variations. A systematic evolutionary analysis comparing the GH17 subfamily in *Arabidopsis thaliana* and tomatoes based on protein sequence homology revealed that these members cluster according to sequence similarity, indicating the conservative nature of the GH17 subfamily across both species. The expansion of the GH17 subfamily in the plants showed significant differences in the number of members among mosses, monocots, and dicots. Additionally, the study highlighted the conservation of the GH17 domain throughout the evolutionary process [9]. 

GH17 genes were reported to be involved in the stress and hormone responses. In Panax ginseng, the homologous gene of *GH17*, *PnGlu1*, significantly improved *Fusarium* resistance in tobacco [10]. The proteomic analyses showed that *GH17* responded to salt stress in ginseng leaves. The overexpression of *GH17* in Arabidopsis enhanced plant salt tolerance. In rice, endo-β-1,3-glucanase’s expression was upregulated by ABA and drought stress [11,12]. In our study, a cis-acting element analysis of the promoters of tomato GH17s revealed that the promoters of the tomato GH17 family members contain hormone-responsive, growth-developmental, and adversity-stress-responsive elements, suggesting that these family members may play significant roles in these response processes.

The GH17 family was also reported to be crucial for plant reproductive development. It was found that the β-1,3-glucanase activity was significantly different in fertile and sterile lines of wheat, indicating its role in wheat male sterility [17]. GH17 members are linked to callose formation in anthers. The overexpression of the β-1,3-glucanase gene causes callose deficiency, leading to male sterility [14]. In Lilium, β-1,3-glucanase activity is associated with callose deficiency [15], while in *Arabidopsis* thaliana, β-1,3-glucan is essential for pollen wall growth [18]. The *AtA6* gene in *Arabidopsis*, encoding β-1,3-glucanase, is a downstream target gene of MYB80, localized in the tapetum and is vital for tapetum degradation [19,20]. *AtA6* was expressed during anther development and was upregulated in the male sterility mutant *cdm1* [22]. In our study, *SlA6* was the downstream target gene of SlHB8, which is localized in the tapetum and is vital for tapetum degradation [21]. The *SlA6* is upregulated in the SlHB8 overexpression lines [21]. A loss of function of the *SlA6* mutant showed reduced pollen activity and pollen tuber germination rate, as well as fewer seeds in the fruit, indicating its important role in pollen development (Figure 7). Whether this phenotype is related to tapetum degradation needs to be further investigated. In Chinese cabbage, the *AtA6* homologs *Bra032758* and *Bra037057* displayed reduced expression during the pollen tetrad formation but increased by 30-fold and 60-fold, respectively, during mononuclear microspore development [23]. In our study, we found that *SlA6* exhibited a specific expression pattern in flower organs, peaking at a bud length of 2 mm. Furthermore, the qRT-PCR data indicated that its highest expression occurs at a bud length of 4 mm at the tetrad stage. Both transcriptome sequencing and qRT-PCR data suggest that *SlA6* plays a crucial role in tomato flower and pollen development, particularly from the tetrad to uninucleate microspore stages.

Previous research across species, such as *Arabidopsis*, *Chinese cabbage*, and *rice*, has demonstrated that a disruption or downregulation of the β-1,3-glucanase genes leads to callose degradation, ending in male sterility [19,24]. In this study, using CRISPR/Cas9 technology on the Micro-Tom tomato genome led to the successful knockout of the *SlA6* gene-producing homozygous mutant *Sla6*. Significant reductions were observed between this mutant and wild-type (WT) concerning the pollen viability, pollen tube germination rate, and per-fruit seed count, indicating that *SlA6* influences pollen development, which is consistent with its high expression in tomato buds and its variation during pollen development. However, a higher total soluble solid (TSS) value was noted in *Sla6* compared with the WT. The increase in total soluble solids (TSSs) may be due to the reduction in seed number, which is similar to parthenocarpic fruits. Analysis of the gene expression patterns revealed similar patterns and levels between *SlA6* and *SlGH37*. Furthermore, the phylogenetic analysis indicated that both genes belong to the same branch, implying similar functions. Therefore, the retained viability of *Sla6*’s pollen could be linked to the functional redundancy between *SlGH37* and *SlA6*.

## 4. Materials and Methods

### 4.1. Plant Materials

The Micro-Tom (MT) tomato, provided by the University of Toulouse in France, was chosen for this study. This small dwarf model crop is characterized by rapid growth, a compact genome, and high efficiency in genetic transformation, making it ideal for berry-type model crop research. All plants were cultivated in the artificial greenhouse at the Horticultural College of South China Agricultural University under a 16 h light/8 h dark photoperiod with an ambient temperature maintained at 25 °C. The planting medium consisted of a 2:1 mixture of peat and vermiculite and was placed in pots with a diameter of 10 cm. During their growth period, the plants were fertilized with Huabao 2 granular fertilizer dissolved in water.

### 4.2. Identification of GH17 Family Members

Using the conserved domain (PF00332) of the GH17 family, sequences from the tomato genome with a *p*-value of less than 0.05 were identified using Hmmersearch software (version 3.2.1). Concurrently, tomato sequences highly homologous to the *Arabidopsis thaliana* GH17 family members and with a *p*-value of less than 0.05 were identified via BLAST software (version 2.16.0). By analyzing the overlap between these two screening results, the GH17 subfamily member genes in tomato were identified. The tomato genome version used was SL4.0, with the gene annotation file version ITAG4.1. The CDS and protein sequences of the Arabidopsis GH17 subfamily members were sourced from the TAIR Arabidopsis genome database. Information on other species mentioned was obtained from the JGI database (https://phytozome-next.jgi.doe.gov/, accessed on 2 June 2021) 

### 4.3. Structural and Evolutionary Analyses of GH17 Family Members

The ProtParam tool was used to analyze the physicochemical properties of the candidate genes, such as the amino acid number, molecular weight, theoretical pI, and instability index [25]. The online tool MEME was employed to analyze the conserved motifs of the candidate genes. The conserved domains of the protein sequences of the candidate genes were analyzed using NCBI’s Batch CD-Search and SMART domain analysis tools [26]. The cis-elements in the promoter region 2000 bp, upstream of the 5‘UTR of the candidate genes, were predicted using the PlantPAN 4.0 (http://plantpan.itps.ncku.edu.tw/plantpan4/, accessed on 2 June 2024) [27]. MUSCLE (version 3.7) and trimAL software (version 1.5.0) were used for the multiple sequence alignment and trimming of the protein sequences of the candidate genes and homologous genes from Arabidopsis. An ML (maximum likelihood) tree was constructed using IQ-tree software for the phylogenetic analysis. Finally, the evolutionary tree, gene structure, conserved motifs, and conserved domains of the candidate genes were visualized using the Gene Structure View (Advanced) function of the TBtools software (version 2.1.1.9) [28].

### 4.4. Expression Pattern Analysis of GH17 Family Members

The spatiotemporal expression patterns of the GH17 family members were analyzed using the transcriptome data from three different transcriptomes as follows: the root (R), stem (S), leaf (L), and flower (F) samples from tomatoes at different stages (BioProject: PRJNA559888) [29]; pollen sample transcriptome sequencing data (CNCB: CRA001723) [30]; and flower bud sample transcriptome sequencing data [21].

The samples F30, L30, R30, and S30 are the flowers, leaves, roots, and stems collected 30 days after germination; samples F45, L45, R45, and S45 are the flowers, leaves, roots, and stems collected 45 days after germination; samples L85, F85, and S85 are the leaves, roots, and stems collected 85 days after germination; and samples 10DPA, 20DPA, IMG, MG, Br, Br3, Br7, Br10, and Br15 samples are the fruit samples collected at 55, 65, 75, 80, 85, 92, 95, and 100 days after germination, respectively. The bud-2mm, bud-4mm, and bud-8mm samples are the tomato anthers collected when the length of the bud was 2 mm (microspore mother cell stage), 4 mm (tetrad stage), and 8 mm (mature pollen stage). Pollen-tetrad, pollen-meiotic, and pollen-mature are the pollen samples at different periods of pollen development.

### 4.5. Expression Pattern Analysis of SlA6

Wild-type tomato seedlings at a consistent growth stage were selected for the flower bud collection. Flower buds at lengths of 0.5, 1, 1.5, 2, 2.5, 3, 4, 6, and 8 mm, along with fully open flowers from the same day were harvested. Tomato seedlings of one month old were treated with 1/2 MS medium supplemented with 100 μM ABA, 100 μM GA3, 100 μM IAA, 100 μM MeJA, or 40% ethylene as the controls. Leaf samples were collected at 0, 1, 6, and 12 h after treatment. Each sample was taken in triplicate, flash-frozen in liquid nitrogen, and stored at −80 °C.

### 4.6. Quantitative Real-Time PCR Analysis

RNA from the above samples was extracted using Omega’s RNA extraction kit. TaKaRa’s PrimeScript TM RT reagent Kit with gDNA Eraser was used to reverse-transcribe the RNA into cDNA at a 1/5 concentration. Real-time fluorescent quantitative PCR primers were designed (Table S3), and their amplification efficiency was assessed. TaKaRa’s TB GreenTM Premix Ex TaqTM II kit was used for real-time PCR with SlUBI used as the reference gene [31]. Real-time data were acquired using the LightCycler 480 high-throughput real-time PCR instrument, and relative quantification analysis was performed using the 2^−△△Ct^ method.

### 4.7. Generating SlA6 Mutants Using CRISPR/Cas9 Technology

The CRISPR/Cas9 gene-editing vector pAGM4723-a6, containing the *SlA6* guide RNA, was introduced into *Agrobacterium GV3101*. The MT tomato underwent genetic transformation through the Agrobacterium-mediated leaf disk method. Following infection of the cotyledon tissues, they were cultured on a 100 mg/L Kanamycin-resistant medium to select the well-differentiated tissue culture seedlings.

### 4.8. Identification of Transgenic Plant Phenotypes

Thirty days post-germination, the *Sla6* mutant and wild-type tomato plants were assessed for height. The stem’s thickness between the first and second nodes was measured, alongside recording the days from germination to anthesis. Five tomato plants at the same developmental stage were chosen, each with 15 preserved flowers; the flowering dates were noted, and other flowers were removed. Fruit counts were conducted 15 days after flowering, and the tomato fruit set rate was calculated. Tomato fruits were harvested seven days after the breaker stage. Ten tomato fruits of consistent size were selected for measuring the fruit weight, seed count per fruit, and total soluble solids content (TSS), followed by statistical analysis.

The total soluble solids content (TSS) was determined using a saccharimeter, and each sample was repeated three times, recorded, and counted.

The pollen viability was assessed using the TTC method. We conducted pollen germination rate experiments in accordance with the protocols established by our laboratory [21]. Fifty seeds from both the *Sla6* mutant and wild-type tomato plants were distributed evenly across dishes, with three replicates each, and then placed in a culture chamber maintained at 95% humidity and 28 °C for seven days. The daily observations involved counting the number of germinated seeds and calculating the germination potential from all of the normal germinations observed within the first four days to ultimately determine the germination rate and index.

### 4.9. Statistical Analysis

All experimental data from this study were analyzed using SPSS statistical software (version 16). Data sets that conformed to or closely approximated a normal distribution were compared using t-tests to assess the significance. Standard deviation (SD) indicated any errors, with the * representing *p* < 0.05, ** representing *p* < 0.01, and *** denoting *p* < 0.001 significance levels.

## Figures and Tables

**Table 1:** Analysis of physicochemical properties of tomato GH17 family members.

Name	ID	Protein Length/bp	Molecular Weight/Da	Theoretical pI	Instability Coefficient	Aliphatic Amino Acid Index	Hydrophilicity Coefficient
SlGH17-1	Solyc01g008610	348	39.23	5.78	34.29	93.3	−0.108
SlGH17-2	Solyc01g008620	336	37.45	6.09	38.35	87.41	−0.289
SlGH17-3	Solyc01g059965	360	39.72	7.84	46.03	89.08	−0.156
SlGH17-4	Solyc01g060020	360	39.72	7.84	46.03	89.08	−0.156
SlGH17-5	Solyc01g109570	473	50.30	4.95	38.16	81.71	−0.079
SlGH17-6	Solyc02g070450	399	44.69	6.12	41.81	91.45	0.017
SlGH17-7	Solyc02g080660	413	45.27	5.46	41.71	83.56	−0.105
SlGH17-8	Solyc02g086700	362	40.82	5.9	32.19	90.19	−0.087
SlGH17-9	Solyc03g025650	337	36.56	5	29.84	103.83	0.159
SlGH17-10	Solyc03g058450	502	54.68	5.68	35.14	78.25	−0.217
SlGH17-11	Solyc03g082900	378	41.68	5.15	31.76	107.28	0.226
SlGH17-12	Solyc04g007910	505	54.64	5.33	40.92	90.2	0.055
SlGH17-13	Solyc04g011720	462	52.12	7.06	30.75	85.82	−0.187
SlGH17-14	Solyc04g011730	466	52.98	8.73	32.12	83.03	−0.248
SlGH17-15	Solyc04g015190	493	53.91	5.9	40.06	94.89	0.126
SlGH17-16	Solyc04g051590	476	52.85	6.66	31.22	83.57	−0.097
SlGH17-17	Solyc05g006210	494	55.02	5.69	39.82	89.03	−0.016
SlGH17-18	Solyc05g015160	470	53.50	6.72	29.86	82.13	−0.233
SlGH17-19	Solyc05g015170	420	47.84	6.46	26.5	81.64	−0.199
SlGH17-20	Solyc05g025500	482	52.71	6.32	31.91	83.36	−0.1
SlGH17-21	Solyc05g054440	526	57.62	9.13	39.78	76.16	−0.227
SlGH17-22	Solyc06g073710	420	46.91	8.67	38.68	97.79	−0.027
SlGH17-23	Solyc06g076170	449	49.37	4.93	29.93	82.09	−0.041
SlGH17-24	Solyc07g005330	459	50.92	9.05	37.72	77.52	−0.25
SlGH17-25	Solyc07g008150	390	42.83	8.73	26.21	93.1	−0.051
SlGH17-26	Solyc07g017730	509	56.33	7.19	33.18	88.82	−0.092
SlGH17-27	Solyc07g049370	468	51.32	6.72	33.84	81.71	−0.162
SlGH17-28	Solyc08g074390	489	52.95	5.76	32.4	85.36	−0.088
SlGH17-29	Solyc08g083310	424	45.63	8.05	30.06	91.32	0.03
SlGH17-30	Solyc09g057630	464	51.04	9.28	31.17	85.93	−0.136
SlGH17-31	Solyc10g078510	479	53.38	5.43	30.27	84.49	−0.073
SlGH17-32	Solyc10g079860	344	37.80	9.42	46.67	93.02	−0.193
SlGH17-33	Solyc11g012030	493	55.20	4.87	38.07	90.77	−0.057
SlGH17-34	Solyc11g065280	338	37.39	4.84	45.4	88.85	−0.212
SlGH17-35	Solyc11g068440	380	42.56	6.06	27.47	90.11	−0.056
SlGH17-36	Solyc11g071520	383	42.56	9.15	38.18	93.94	0.025
SlGH17-37	Solyc11g072230	411	45.19	5.38	37.27	98.69	0.209
SlGH17-38	Solyc12g008580	496	53.95	5.91	34.82	79.31	−0.073
SlGH17-39	Solyc12g014420	480	51.46	5.59	39.02	82.9	−0.149
SlGH17-40	Solyc12g019890	475	52.35	4.89	32.02	81.96	−0.052
SlGH17-41	Solyc12g040860	456	50.39	6	29.34	84.87	−0.134
SlGH17-42	Solyc12g055840	422	45.54	6.52	32.48	88.48	0.006
SlGH17-43/SlA6	Solyc12g098560	459	51.89	5.09	39.87	85.62	−0.22

## Data Availability

All data generated or analyzed during this study are included in this published article and its Supplementary Information files.

## Supplementary Materials

The following supporting information can be downloaded at: https://www.mdpi.com/article/10.3390/plants13172443/s1. Figure S1. Positive identification of T0 generation *SlA6* plants; Figure S2. Analysis of covariance in tomato and other species; Table S1. Number of introns and exons of SlGH17 family members; Table S2. GH17 family members from different plant species; Table S3. Quantitative PCR primers.
