# Supplementary material for: Genome-Wide Identification of GH17s Family Genes and Biological Function Analysis of SlA6 in Tomato
Source: Plants (Basel). 2024 Sep 1;13(17):2443. doi: 10.3390/plants13172443 (PMC11397118; doi:10.3390/plants13172443)
Supplement: Supplementary file 1 [file plants-13-02443-s001.zip › plants-3148104-supplementary.pdf]

Supplementary Information

Article title: Genome-Wide Identification of GH17s Family Genes and Biological Function Analysis of SlA6 in Tomato

Authors: Da Chen[1†], Zaohai Zeng[1†], Canye Yu[1], Huimin Hu[1], Yuxiang Lin[1,3], Caiyu Wu[1], Yinghua Yang[1], Qiuxiang Zhong[1], Xinyue Zhang[1], Caihong Huang[1], Yiwen Yao[1], Zhengkun Qiu[1], Xiaomin Wang [4], Rui Xia[1], Chongjian Ma[2], Riyuan Chen[1], Yanwei Hao[1]*, Hongling Guan[1,2]*

The following Supporting Information is available for this article:

Figures S1-S2

Tables S1-S3

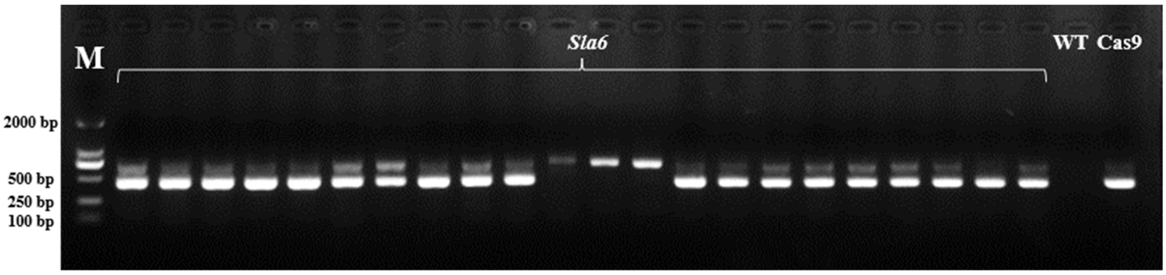

Figure S1. Positive identification of T0 generation SlA6 plants.

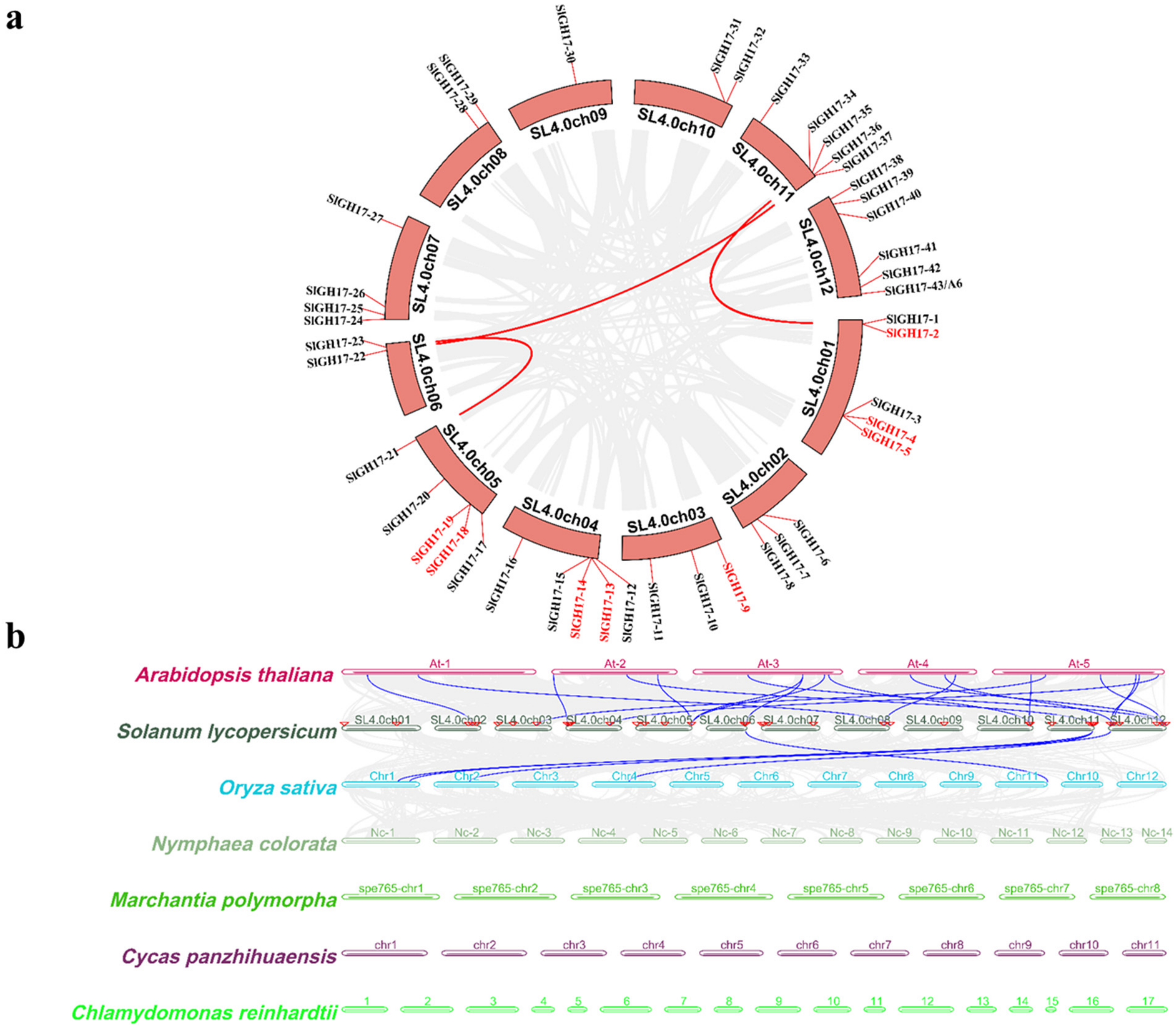

Figure S2. Analysis of covariance in tomato and other species.

a Analysis of covariance in tomato； b Covariance analysis of tomato and other species.

Table S1 Number of introns and exons of SlGH17s family members.

Gene name	Gene ID	exon number	intron number
SlGH17-1	Solyc01g008610	2	1
SlGH17-2	Solyc01g008620	2	1
SlGH17-3	Solyc01g059965	2	1
SlGH17-4	Solyc01g060020	2	1
SlGH17-5	Solyc01g109570	6	5
SlGH17-6	Solyc02g070450	3	2
SlGH17-7	Solyc02g080660	5	4
SlGH17-8	Solyc02g086700	2	1
SlGH17-9	Solyc03g025650	2	1
SlGH17-10	Solyc03g058450	2	1
SlGH17-11	Solyc03g082900	2	1
SlGH17-12	Solyc04g007910	4	3
SlGH17-13	Solyc04g011720	3	2
SlGH17-14	Solyc04g011730	3	2
SlGH17-15	Solyc04g015190	2	1
SlGH17-16	Solyc04g051590	3	2
SlGH17-17	Solyc05g006210	2	1
SlGH17-18	Solyc05g015160	3	2
SlGH17-19	Solyc05g015170	4	3
SlGH17-20	Solyc05g025500	2	1
SlGH17-21	Solyc05g054440	3	2
SlGH17-22	Solyc06g073710	3	2
SlGH17-23	Solyc06g076170	3	2
SlGH17-24	Solyc07g005330	3	2
SlGH17-25	Solyc07g008150	3	2
SlGH17-26	Solyc07g017730	2	1
SlGH17-27	Solyc07g049370	3	2
SlGH17-28	Solyc08g074390	2	1
SlGH17-29	Solyc08g083310	2	1
SlGH17-30	Solyc09g057630	3	2
SlGH17-31	Solyc10g078510	4	3
SlGH17-32	Solyc10g079860	2	1
SlGH17-33	Solyc11g012030	2	1
SlGH17-34	Solyc11g065280	1	0
SlGH17-35	Solyc11g068440	3	2
SlGH17-36	Solyc11g071520	3	2
SlGH17-37	Solyc11g072230	1	0
SlGH17-38	Solyc12g008580	4	3
SlGH17-39	Solyc12g014420	2	1
SlGH17-40	Solyc12g019890	2	1
SlGH17-41	Solyc12g040860	2	1
SlGH17-42	Solyc12g055840	2	1
SlGH17-43/SlA6	Solyc12g098560	2	1

Table S2 GH17s family members from different plant species.

Species	α	β	γ	total	Category
Glycine max	106	23	23	152	Eudicots
Populus trichocarpa	96	14	19	129	Eudicots
Solanum tuberosum	42	24	24	90	Eudicots
Malus domestica	40	23	25	88	Eudicots
Citrus sinensis	32	20	10	62	Eudicots
Arabidopsis thaliana	28	11	11	50	Eudicots
Solanum lycopersicum	21	8	14	43	Eudicots
Zea mays	66	15	13	94	Monocots
Ananas comosus	23	8	13	44	Monocots
Oryza sativa	69	23	14	106	Monocots
Sorghum bicolor	39	23	19	81	Monocots
Nymphaea colorata	35	12	17	64	Base angiosperms
Amborella trichopoda	20	12	9	41	Base angiosperms
Cycas panzhihuaensis	44	12	5	61	Gymnospermae
Gnetum montanum	29	8	5	42	Gymnospermae
Marchantia polymorpha	29	1	2	32	Bryophyta
Chara braunii	14	0	0	14	Algae

Table S3 Quantitative PCR primers.

Name	Sequences
q-SlA6-F	GCTGAAACCGGATGGCCT
q-SlA6-R	GCTGGTGTCCCAATGGCT
q-SlUBI-F	CCAAGATCCAGGACAAGGAA
q-SlUBI-R	AAATCAAACGCTGCTGGTCT
